# The Differential Experience of COVID-19 on Asian American Subgroups: The Los Angeles Pandemic Surveillance Cohort Study

**DOI:** 10.1007/s40615-023-01742-y

**Published:** 2023-10-11

**Authors:** Chun Nok Lam, Benjamin Tam, Eric S. Kawaguchi, Jennifer B. Unger, Kevin Hur

**Affiliations:** 1 Department of Emergency Medicine, Keck School of Medicine of USC, 1200 N State Street, Room 1011, Los Angeles, CA 90033 USA; 2 Department of Population and Public Health Sciences, Keck School of Medicine of USC, 1845 N Soto Street, Los Angeles, CA 90032 USA; 3 Caruso Department of Otolaryngology – Head and Neck Surgery, Keck School of Medicine of USC, Los Angeles, USA

**Keywords:** Asian American, COVID-19, Disparities, Testing, Vaccination, Health behaviors

## Abstract

Data from Asian Americans (AsA) are commonly aggregated in research studies and reporting, obscuring the significant differences across AsA subgroups. We investigated the differential experience of AsA subgroups in COVID-19 testing, vaccination, engagement in risky and protective behaviors and mental health status against this infectious disease. We surveyed a representative sample of the Los Angeles County population (*N* = 5500) in April 2021 as part of the Los Angeles Pandemic Surveillance Cohort Study and focused on participants who self-identified as AsA (*N* = 756). There were significant differences across the AsA subgroups, with Koreans, Asian Indians, and Other Asians living in areas with higher COVID-19 mortality rates, and Asian Indians demonstrating the lowest proportion of COVID-19 vaccination. Vietnamese and Koreans had a higher proportion of becoming unemployed during the pandemic. Although the AsA sample on average demonstrated better outcomes than other racial and ethnic groups, the apparent advantages were heterogenous and due to specific subgroups of AsAs rather than AsAs as a whole. The observed differences in COVID-19 measures across AsA subgroups underscore the need to disaggregate AsA data to identify and reduce existing disparities.

**Supplementary Information:**

The online version contains supplementary material available at 10.1007/s40615-023-01742-y.

## Introduction

The Asian American (AsA) population is a large and highly diverse group with individuals tracing their ancestral origins to over 20 different countries. The term “Asian” refers to those with origins in the Far East, Southeast Asia and the Indian subcontinent and includes groups such as Chinese, Hmong, Filipino, Indian, Korean and Pakistani [1]. The AsA population is the fastest growing racial/ethnic group in the USA with an 81% increase from 2000 to 2019, which was higher than the 71% increase in those who identify as Hispanic [2]. As of 2019, there were 22.4 million AsAs with a projected increase to over 46 million by 2060 [2]. Though highly diverse, the majority of AsAs (85%) in the USA identify with one of the six origin groups. These groups include Chinese (24%), Indian (21%), Filipino (19%), Vietnamese (10%), Korean (9%) and Japanese (7%) [3]. California accounts for 6.7 million AsAs, representing 30% of the country’s AsA population [2]. This makes California and its diverse metropolitan centers, such as Los Angeles County, ideal for studies investigating the AsA population [3].

Though there is tremendous diversity in geographic origin, circumstances of immigration, historical context of reception in the USA, language, tradition, and cultural beliefs within the AsA population, studies commonly aggregate the subgroups together into an “Asian race” category without stratification. This often results in the failure to discriminate differences between these unique subgroups. Progress has been made in recent years to distinguish health disparities between these ethnic groups. For example, it has been established that Filipinos have a greater health burden than other AsA subgroups, particularly in chronic conditions such as diabetes mellitus, hypertension and coronary artery disease [4–9]. There also exist differences in access to preventative medical resources such as Papanicolaou tests and mammograms with Korean women being tested less frequently than their AsA peers [10], and differences in health behaviors such as smoking and alcohol use [11]. While we are still developing a better understanding of health disparities within the AsA group, there continues to be a paucity of literature detailing the etiology of these disparities and why they continue to exist [10]. Knowledge of the differences between AsA subgroups in health literacy, healthcare perceptions and attitudes are instrumental in guiding health interventions that address each specific subgroup appropriately.

AsAs broadly share collectivist values as opposed to the Western idealization of individualistic values [12–15]. Those with collectivist values tend to prioritize the family and surrounding community’s needs over one’s own desires, whereas those with individualistic values will be less likely to engage in behaviors which are not self-beneficial. These collectivistic ideals have been shown to affect COVID-19-related health behaviors in Asians/AsAs with higher rates of masking, hand washing, vaccination and trust in health authorities as compared to Westerners. While AsAs generally have collectivistic values, each subgroup is different and assimilation into American culture likely creates a mixture of collectivistic and individualistic values. A better understanding of how these behaviors differ between AsA subgroups can help to identify groups most at risk during a pandemic such as COVID-19.

COVID-19, the infectious disease responsible for a global pandemic, has been highly prevalent in the USA, with over 92 million confirmed cases and over a million deaths as of August 2022 [16]. For AsAs, studies have reported a higher case fatality rate than Hispanic and non-Hispanic Black Americans, and elevated racially motivated violence and discrimination toward AsAs [17, 18]. A study by Chin et al. suggested their high infection outcomes can be due to their overrepresentation in the frontline and high-contact essential work force, and high rates of living in multigenerational households [19]. AsAs experienced not only the physical health challenges of a global pandemic, but also increased feelings of depression and isolation with a sense of heightened vigilance due to fear of racist harassment [20]. As the National Academies of Sciences, Engineering and Medicine highlighted in their 2021 proceeding, due to the growing concerns of the impact of COVID-19 on AsA communities, it is important to harness all available data and develop new data disaggregation strategies to analyze the disproportionate effects of COVID-19 on AsA subgroups [21].

The goal of this study was to describe the unique experiences of AsA subgroups during the COVID-19 pandemic, including variation in testing, vaccination, employment, health behaviors, and mental health status. Study findings will help provide insights to the differential impacts of COVID-19 among AsA subgroups to help develop better public health strategies for each unique community of the AsA population.

## Methods

An online survey was conducted in Los Angeles County using a representative sample recruited from a proprietary database (LRW, A Material Company) between March 22^nd^ and April 24^th^, 2021. A total of 5500 adult participants from all racial and ethnic groups completed the survey and provided informed consent. A detailed description of the recruitment strategy is discussed in Nicholas et al. [22]. Questionnaire items were designed to understand health impacts on Los Angeles County residents during COVID-19. The Los Angeles County Department of Public Health Institutional Review Board approved the study procedures. The survey was available in English and Spanish languages.

### Sample Population

The primary sample included participants of AsA heritage using self-reported data on race and ethnicity. Out of 5500 participants from the original sample, 756 (13.7%) reported AsA heritage. Our analysis focused on 8 AsA subgroups: Chinese, Korean, Filipino, Vietnamese, Japanese, Asian Indian, Mixed Asian, and Other Asians. Other Asians included individuals of South/Southeast Asian origin; they were aggregated into a single group because there were not enough respondents to analyze separately. Participants answered the two-item race and ethnicity question using the Office of Management and Budget Standards [23], with specific AsA subgroups as an add-on item when the Asian race was selected. The proportion of the AsA subgroups in the study was similar to their overall population reported in the 2019 Census in Los Angeles County [24].

Our paper primarily focused on the AsA subgroups, though we also provided data on the larger racial and ethnic groups from our full sample, which included Non-Hispanic White, Non-Hispanic Black, Non-Hispanic Other, Non-Hispanic AsA (aggregate of our AsA subgroup data), and the Hispanic populations.

### Study Measures

We collected demographic characteristics including gender, age, education, household income and employment status. We used self-reported residential ZIP code to convert to the Service Planning Area (SPA) specific to Los Angeles County, and further into three levels of COVID-19 mortality impacted areas (CMIA) based on COVID-19 mortality rates at the SPA level [25]. Low CMIA included SPA 5 and 8; middle CMIA included SPA 1, 2 and 3; and high CMIA included SPA 4, 6 and 7. The employment item asked if participants worked as an essential worker during COVID-19, and the definition of essential workers is described in detail in Nicolas et al., a study published using this dataset [22]. The key COVID-19-related measures included ever-tested and ever-tested positive for COVID-19, COVID-19 vaccination status (with 1 + dose), 6 items related to the practice of risky and protective behaviors in the past 7 days (i.e., having visitors, attending gatherings, going to social places, washing hands, staying 6 feet away, and wearing a facemask), and two mental health questions asking, “In the past 7 days, how worried were you about catching COVID-19” and “In general, how do you rate your mental health, including your mood and your ability to think?” [26]. Response options were dichotomous (yes vs no) or ordinal, as described in the result section.

### Statistical Analysis

We conducted Chi-square tests to determine whether demographic characteristics were statistically different across the AsA subgroups as well as across racial and ethnic groups. Age, gender, education, income, and CMIA were adjusted for in the multivariable logistic regression models for COVID-19 testing and vaccination outcomes and ordinal regression models for risk/protective behaviors [2]. The primary focus of this paper is to test for an overall difference in each of the COVID-19 outcomes (1) across the AsA subgroups and (2) across racial and ethnic groups. To test for an overall difference across the eight AsA subgroups, a 7 degree of freedom likelihood ratio test was conducted. Similarly, we perform a 4 degree of freedom likelihood ratio test to test for an overall difference across five racial and ethnic groups. *P*-values from the corresponding likelihood ratio test are reported. Since our focus was not on estimating differences between certain AsA subgroups or between AsA and other racial and ethnic groups, pairwise comparisons (e.g., comparisons relative to a reference group with an associated odds ratio, 95% confidence interval and *p*-value) were not performed or reported. Due to the exploratory nature of this study, no formal adjustment for multiple comparisons were performed. Statistical analyses were performed using Stata 15 with a two-sided significance level of 0.05.

## Results

### Demographic Characteristics Across AsA Subgroups

Table 1 describes the 756 unique AsA participants, including Chinese (35%), Korean (18%), Filipino (19%), Vietnamese (7%), Japanese (7%), Asian Indian (5%), Mixed Asian (5%), and Other Asians (6%) participants. Among the AsA participants, 50% were male, 45% were 18–34 years old, 78% were college graduate and beyond, 37% had $100,000 or more in annual household income, 32% spoke a language other than English at home, and 29% lived in a high CMIA. Since the pandemic, 18% of AsA participants experienced reduced hours or wages, 11% became unemployed, and 29% worked as essential workers. There were significant differences for all factors across AsA subgroups. Specifically, Koreans (46%), Asian Indians (38%) and Other Asians (44%) reported the highest proportions of living in high CMIA. Other Asians (35%) reported the highest proportion of experiencing reduced hours or wages; Koreans (15%) and Vietnamese (15%) reported the highest proportions of becoming unemployed.

**Table 1:** Demographics characteristics among Asian American subgroups

	Overall *n* = 756 (100%)	Chinese *n* = 262 (35%)	Korean *n* = 136 (18%)	Filipino *n* = 142 (19%)	Vietnamese *n* = 53 (7%)	Japanese *n* = 52 (7%)	Asian Indian *n* = 34 (5%)	Mixed Asian *n* = 34 (5%)	Other Asians *n* = 43 (6%)	*p*-value
Gender										0.02
Male	50%	58%	51%	46%	36%	33%	53%	47%	42%	
Female	50%	42%	49%	52%	64%	67%	47%	50%	58%	
Non-binary	1%	0%	0%	1%	0%	0%	0%	3%	0%	
Age										< 0.01
18–34	45%	44%	41%	45%	58%	23%	62%	74%	37%	
35–49	38%	42%	45%	42%	26%	21%	24%	21%	49%	
50–64	13%	11%	13%	13%	13%	37%	9%	6%	9%	
≥ 65	4%	4%	1%	1%	2%	19%	6%	0%	5%	
Education										< 0.01
High school or below	6%	4%	8%	6%	11%	2%	9%	12%	9%	
Some college	16%	10%	13%	21%	21%	29%	18%	18%	28%	
College graduate	53%	57%	51%	58%	49%	42%	47%	53%	37%	
Post-graduate	25%	29%	28%	15%	19%	27%	26%	18%	26%	
Annual household income										0.06
Under $50,000	24%	20%	27%	30%	36%	15%	26%	24%	23%	
$50,000–$99,999	31%	29%	27%	37%	36%	33%	32%	26%	35%	
>= $100,000	37%	44%	42%	27%	19%	38%	32%	41%	37%	
Prefer not to answer	7%	8%	4%	6%	9%	13%	9%	9%	5%	
Language spoken at home										< 0.01
Only English	42%	42%	33%	43%	28%	75%	44%	44%	51%	
Mostly English	25%	26%	32%	25%	28%	10%	24%	26%	16%	
English and another language	23%	23%	22%	28%	25%	12%	26%	21%	26%	
Mostly another language	8%	8%	11%	4%	19%	4%	3%	9%	7%	
Only another language	1%	3%	2%	0%	0%	0%	3%	0%	0%	
Residential region^a^										< 0.01
Low CMIA	27%	28%	26%	23%	19%	46%	24%	29%	30%	
Middle CMIA	43%	52%	28%	48%	58%	29%	38%	47%	26%	
High CMIA	29%	20%	46%	29%	23%	25%	38%	24%	44%	
Change in employment status										< 0.01
Not employed^b^	10%	10%	4%	11%	15%	27%	12%	6%	7%	
No change	61%	64%	67%	61%	51%	56%	56%	59%	49%	
Reduced hours/wages	18%	18%	13%	18%	19%	13%	21%	24%	35%	
Became unemployed	11%	8%	15%	11%	15%	4%	12%	12%	9%	
Essential workers	29%	26%	26%	40%	25%	17%	35%	24%	30%	0.03

^a^Based on Service Planning Area in Los Angeles County using self-reported ZIP code

^b^Not employed since the pandemic began — includes homemaker, student, retired, and unable to work

*CMIA*, COVID-19 mortality Impacted Areas

### COVID-19 Testing and Positivity

**Fig. 1:**
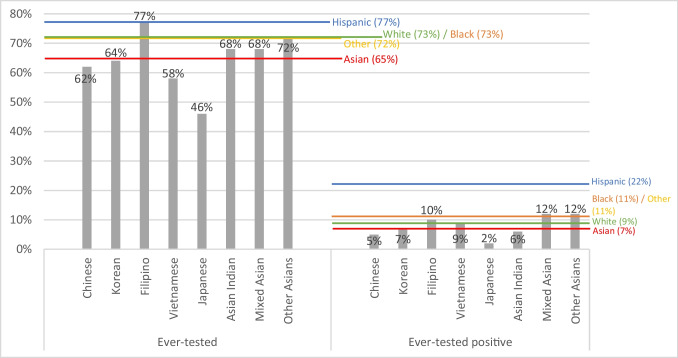
Sample proportion of COVID-19 ever-testing and positivity. For ever-tested, the likelihood ratio test *p*-value across Asian American subgroups is *p* < 0.01, and the *p*-value across the racial and ethnicity groups is *p* < 0.01. For ever-tested positive, the *p*-value across Asian American subgroups is *p* = 0.57, and the *p*-value across the racial and ethnicity groups is *p* < 0.01

Overall, 65% of AsAs had been tested for COVID-19. There was a significant difference in the proportion of being ever tested across AsA subgroups (*p* < 0.01). Filipinos (77%) and other Asians (72%) showed the highest ever-tested proportions for COVID-19, while Japanese (46%) showed the lowest. Among the AsA participants, 7% reported ever-tested positive for COVID-19. However, there was no significant difference across the AsA subgroups (Fig. 1).

### COVID-19 Vaccination Status

**Fig. 2:**
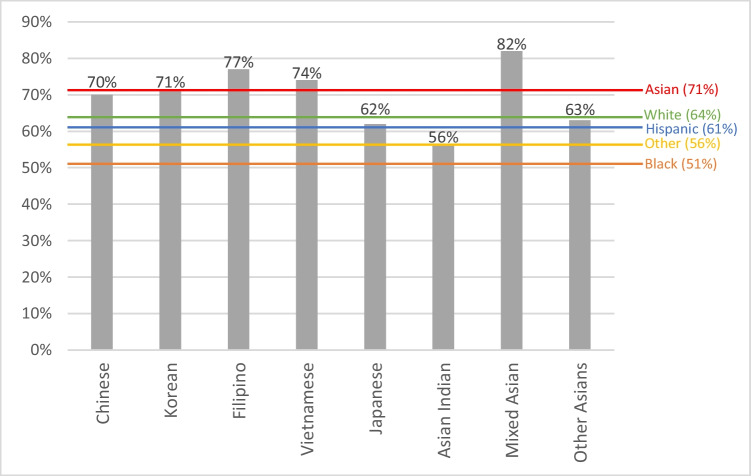
Sample proportion of COVID-19 vaccination uptake. The likelihood ratio test *p*-value across Asian American subgroups is *p* = 0.01, and the *p*-value across the racial and ethnicity groups is *p* < 0.01

Figure 2 demonstrates the COVID-19 vaccine uptake across AsA subgroups. Overall, 71% of AsA received at least 1 dose of COVID-19 vaccine. The unadjusted *p*-value was 0.11, suggesting no significant difference across AsA subgroups However, significant differences across AsA subgroups were identified when controlling for differences in demographic characteristics (*p* = 0.01). Results from the descriptive statistics showed that Mixed Asians (82%) and Filipinos (77%) had the highest proportions of being vaccinated, while Japanese (62%) and Asian Indians (56%) reported the lowest.

### Risky and Protective Behaviors

For risky behaviors, in the past 7 days 35% of AsA participants reported having visitors to their residence (once: 22%, 2 or 3 times: 10%, 4 or more times: 3%) (Supplementary information C). There was no significant difference across the AsA subgroups (Supplementary information A). For attending a large gathering with more than 10 people, 15% of AsA participants (once: 12%, 2 or 3 times: 2%, 4 or more times: 1%) reported practicing the behavior. There was a significant difference across AsA subgroups (*p* = 0.03). Filipino (23%) and mixed Asian (21%) had the highest proportion of reporting once, 2 or times and 4 or more times combined, while Chinese (10%) and Japanese (10%) had the lowest. For “gone out to a bar, club, or other social venues”, 17% of AsA participants (once: 10%, 2 or 3 times: 6%, 4 or more times: 1%) reported practicing the behavior. There was no significant difference across the AsA subgroups.

For protective behaviors, in the past 7 days 97% of AsA participants reported washing hands or using hand sanitizer frequently. For staying 6 feet away from non-household members, 83% reported practicing the protective behavior. For wearing a mask when in the presence of others, 98% reported practicing the protective behavior. There was no significant difference across the AsA subgroups for all three protective behaviors.

### Mental Health Outcomes

When asked how worried they were about catching COVID-19, AsAs reported not at all (36%), slightly (37%), moderately (19%), very worried (5%), and extremely worried (3%) (Supplementary information D). For mental health wellbeing, responses were poor (5%), fair (18%), good (31%), very good (31%), and excellent (16%). There was no significant difference across the AsA subgroups in both mental health measures (Supplementary information B).

### Study Outcomes across Racial and Ethnic Groups

We incorporated all study data (*n* = 5500) comparing racial and ethnic groups to provide a reference point where the non-Hispanic AsA participants and each AsA subgroup contrasted with Hispanic, Non-Hispanic White, Non-Hispanic Black, and Non-Hispanic Other populations.

For demographic characteristics (Table 2), there was a significant difference across racial and ethnic groups in all factors presented, including age, gender, education, household income, home language spoken, and residential regions impacted by COVID-19 (*p* < 0.01). For residential regions, non-Hispanic Black (51%) and Hispanic (48%) samples had the highest proportions of living in high CMIA, while non-Hispanic AsA (29%) and non-Hispanic White (26%) samples had the lowest. However, specific AsA subgroups including Koreans (46%) and Other Asians (44%) far exceeded the overall AsA average and were more similar to the non-Hispanic Black and Hispanic samples in terms of how COVID-19 mortality rate affected their residential regions.

**Table 2:** Demographics characteristics across racial and ethnic groups

	Total *n* = 5500 (100%)	Hispanic *n* = 2355 (43%)	Non-Hispanic White *n* = 1682 (31%)	Non-Hispanic Black *n* = 468 (9%)	Non-Hispanic Asian *n* = 756 (14%)	Non-Hispanic Other *n* = 239 (4%)	*p*-value
Gender							< 0.01
Male	45%	47%	41%	42%	50%	41%	
Female	55%	53%	59%	57%	50%	57%	
Non-binary	1%	1%	0%	0%	1%	1%	
Age							< 0.01
18–34	37%	46%	24%	25%	45%	38%	
35–49	33%	32%	31%	33%	38%	43%	
50–64	21%	15%	31%	33%	13%	18%	
≥ 65	8%	7%	13%	9%	4%	2%	
Education							< 0.01
High school or below	17%	29%	6%	12%	6%	6%	
Some college	26%	30%	21%	32%	16%	31%	
College graduate	40%	29%	47%	41%	53%	45%	
Post-graduate	18%	11%	25%	15%	25%	18%	
Annual household income							< 0.01
Under $50,000	36%	45%	27%	41%	24%	33%	
$50,000– $99,999	29%	28%	29%	36%	31%	29%	
>= 100,000	26%	16%	38%	19%	37%	22%	
Prefer not to answer	8%	11%	6%	4%	7%	15%	
Language spoken at home							< 0.01
Only English	54%	26%	85%	93%	42%	75%	
Mostly English	17%	23%	8%	5%	25%	16%	
English and another language	21%	37%	6%	2%	23%	7%	
Mostly another language	5%	8%	1%	0%	8%	1%	
Only another language	3%	5%	0%	0%	1%	1%	
Residential region^a^							< 0.01
Low CMIA	23%	16%	29%	27%	27%	26%	
Middle CMIA	38%	36%	45%	22%	43%	36%	
High CMIA	39%	48%	26%	51%	29%	38%	
Change in employment status							< 0.01
Not employed^b^	14%	16%	14%	12%	10%	10%	
No change	47%	44%	45%	46%	61%	47%	
Reduced hours or wages	23%	23%	25%	24%	18%	23%	
Became unemployed	16%	17%	16%	17%	11%	19%	
Essential workers	29%	36%	21%	29%	29%	29%	< 0.01

^a^Based on Service Planning Area in Los Angeles County using self-reported ZIP code

^b^Not employed since the pandemic began — includes homemaker, student, retired, and unable to work

*CMIA*, COVID-19 mortality impacted areas

For COVID-19 testing and positivity (Fig. 1), there was a significant difference across racial and ethnic groups (*p* < 0.01). Hispanics (77%) reported the highest proportion of being ever tested for COVID-19, while non-Hispanic AsA (65%) reported the lowest. However, Filipinos (77%) reported a proportion similar to the Hispanic sample compared its other AsA counterparts. For COVID-19 positivity, the Hispanic sample (22%) reported the highest proportion of ever-tested positive, with non-Hispanic Black (11%) and non-Hispanic Other (11%) samples following. For AsA subgroups, Other Asians (12%), mixed Asians (12%) and Filipinos (10%) reported higher proportions within the subgroups, but lower than the Hispanic sample.

For COVID-19 vaccine uptake (Fig. 2), there was a significant difference across racial and ethnic groups (*p* < 0.01). Non-Hispanic AsA sample (71%) reported the highest proportion of being vaccinated against COVID-19, while non-Hispanic Black sample (51%) the lowest. Among AsA subgroups, Asian Indians (56%) reported the lowest proportion of being vaccinated, similar to the non-Hispanic Black sample (51%).

For risky behaviors, there were significant differences across racial and ethnic groups (*p* < 0.01) (Supplementary information C). The AsA sample reported less engagement in risky behaviors. For protective behaviors, hand washing and mask wearing showed significant differences (*p* < 0.01). For mental health measures, there was a significant difference on self-reported mental health status (*p* < 0.01), with the non-Hispanic Other (28%) and Hispanic (25%) samples showed a higher reporting of poor and fair responses, while the non-Hispanic White (17%) and non-Hispanic Black (18%) samples reporting the lowest (Supplementary information D). The AsA subgroups showed no significant differences in these areas.

## Discussion

Our study presented AsA subgroup data to determine how the COVID-19 pandemic differentially affected these populations. Differences existed in demographic characteristics, COVID-19 testing, COVID-19 vaccine uptake and risky behaviors. Even though AsAs overall showed better outcomes than other racial and ethnic groups, the apparent advantages for the AsA population were due to specific subgroups rather than all AsAs. Koreans, Asian Indians, and Other Asians lived in areas with higher COVID-19 mortality rates, and Asian Indians demonstrated the lowest proportion of COVID-19 vaccination. Vietnamese and Koreans had a higher proportion of becoming unemployed during the pandemic. The nuances provided by our current data allow better understanding of the discrepancies that exist within the AsA subgroups.

A demographic difference of interest is the high proportion of certain AsA subgroups, specifically Koreans and Other Asians, living in high CMIA. While these proportions were slightly below those of Hispanic and non-Hispanic Black participants, they far exceeded the overall AsA proportion and highlight the flaws of aggregating AsA subgroup data. The high CMIA, including SPA 4 (Metro/downtown), SPA 6 (South Los Angeles) and SPA 7 (East Los Angeles), make up three of the four lowest median household income levels among the 8 SPAs, reflecting the known inverse relationship between COVID-19 infection and household income level [27–29]. Differences in employment changes also reflect how AsA subgroups work in different industries, such as frontline and high-contact essential workforce and thus can be affected by public health emergencies in different ways. It is challenging to interpret this data given the continuing lack of data disaggregation in national surveys such as the Current Population Survey (conducted by the Bureau of Labor Statistics) which, as of August 2022, continues to aggregate AsA labor data [30]. Notably, this disparity in employment changes is not due primarily to any difference in employment in essential industries work force within AsA subgroups in our data, but possibly a characteristic specific to the Los Angeles County population. However, there is a lack of data on whether or not specific AsA subgroups are disproportionally employed in essential industries [31]. This is an area for future research as this can help predict which AsA subgroups may be disproportionally affected in future public health crises.

Significant differences were seen in COVID-19 vaccination across AsA subgroups with Asian Indians reporting the lowest vaccination uptake. Cultural beliefs may play a role. Park et al. performed a national survey of AsAs on vaccine perceptions and reported high vaccine hesitancy in Korean Americans which is not clearly reflected in our data [32]. There is a paucity of data regarding Asian Indians. However, there is some evidence that Asian Indians in their home country are reluctant to be vaccinated with 37% reporting being unsure about getting vaccinated or refusing vaccination [33]. Unique cultural beliefs may play a strong role in predicting vaccine hesitancy. In our study, Japanese participants also had low vaccine uptake despite making up a higher percentage of older adults compared to other subgroups. It is unclear why this occurred as there is a known association between increasing age and vaccine acceptance [31]. Even as we controlled for age in the regression model, the Japanese participants were least likely to be vaccinated. They were also the least likely to be tested for COVID-19, had the highest proportion living in the low CMIA, and the lowest employment in essential industries. Japanese American in our sample may have skewed wealthy and possibly did not feel at risk of being infected with COVID-19, as supported by their lower proportion of reporting being worried of catching the virus in Supplementary information B, thus discouraging them from being tested or vaccinated. Yi et al. found that high income Japanese Americans were more likely to work from home during the pandemic compared to most other AsA subgroups [34]. This may have contributed to a lower willingness to be vaccinated and a decreased need to be tested due to their lower potential for exposure to COVID-19. Another possible explanation is that our Japanese American cohort appears to be well assimilated into American culture with 75% reporting speaking only English at home, easily the highest among all AsA subgroups in our sample, and 0% reporting speaking only another language. Given the racial differences in COVID-19-related health behaviors, it is possible that our Japanese cohort fits somewhere in the middle of the spectrum and displays some behaviors more consistent with AsAs broadly but also some more often seen in Non-Hispanic White Americans [12, 13].

In terms of risky and protective behaviors, we only found a significant difference between AsA subgroups in attending large gatherings. There were no significant findings among the three protective behaviors. This suggests that behavior patterns remain largely uniform across the AsA subgroups. A possible explanation could be the collectivist culture that most AsAs are raised in. Collectivist culture is broadly defined as one in which the group or community is favored over the self and the individual must sacrifice personal desires in order to provide for the general society [35]. This has significant implications for infectious diseases such as COVID-19 in which individuals who are generally not vulnerable to severe infection (young and healthy) must sacrifice for those who are at high risk of severe illness. While certainly not all AsAs have a collectivist mindset, it is generally accepted that Asian cultures are collectivist, as opposed to individualist [36]. Mask wearing is a protective behavior not only for oneself, but also for others nearby. Lu et al. found that collectivism was predictive of mask usage by Americans in the era of COVID-19 [37]. Given that most Asian cultures are predominately collectivist, it is unsurprising that AsAs display relatively low engagement in risky behaviors but high in protective behaviors.

### Limitations

This study has several limitations. First, although our study was performed in a large and diverse metropolitan area, it only captures the Los Angeles County population and thus may not adequately represent AsAs across the country. Second, the sample size for several subgroups (i.e., Asian Indians and Mixed Asians) were smaller relative to others. While their sample size is adequate from an analytic standpoint, it is subject to selection bias for measures of rare events such as testing positive for COVID-19 in the early phase of the pandemic. Targeted recruitment of these subgroups may increase participation in future surveys. Third, it was an online survey and mostly completed in English. It may yield a selection bias toward tech knowledgeable and more acculturated individuals. Future studies can conduct household sampling to increase recruitment of non-English speaking participants who may have different experiences with COVID-19. Finally, our survey relied on self-reported COVID-19 vaccination, testing and infection status, which could not be verified with medical charts for confirmation.

### Future Directions

A national survey may provide data that better represent the AsA subgroups in the USA. Increasing the sample size for each AsA subgroup will allow further diving into specific pairwise differences. Future studies may also evaluate how the AsA subgroups fare in different aspects of COVID-19 such as quarantine/isolation behaviors, vaccine booster administration, and use of vaccine waivers as differences may exist. In addition, developing a study cohort of AsA subgroups will allow longitudinal assessment of their unique experience over time. This paper highlights the need to disaggregate data among AsA subgroups when possible. Previous studies may have missed significant differences between the AsA subgroups by not disaggregating data and this can lead to inequitable treatment of AsAs. This data is essential to guide culturally appropriate discussions and focus our treatment appropriately.

## Conclusion

The COVID-19 pandemic differentially affected AsA subgroups in COVID-19 testing, vaccine uptake and engagement in risky behaviors. While aggregate data may suggest that AsAs have better outcomes compared to other racial and ethnic groups, findings on specific AsA subgroups demonstrate similarities to Hispanic and non-Hispanic Black populations, which are known to be disproportionally affected by the pandemic. Our study data provides evidence to support that each AsA subgroup is unique, from demographics to COVID-19 experience, and should be accounted for independently in future study and data consideration.

## Supplementary Information

Below is the link to the electronic supplementary material.

Supplementary file1 (XLSX 20 KB)

## Data Availability

The dataset generated during the current study is not publicly available as it contains proprietary information from a partnering marketing research company. Information on how to obtain access is available from the corresponding author on request.
